# Grain Size Associated Genes and the Molecular Regulatory Mechanism in Rice

**DOI:** 10.3390/ijms23063169

**Published:** 2022-03-15

**Authors:** Hongzhen Jiang, Anpeng Zhang, Xintong Liu, Jingguang Chen

**Affiliations:** 1 School of Agriculture, Shenzhen Campus of Sun Yat-sen University, Shenzhen 518107, China; jianghzh23@mail.sysu.edu.cn (H.J.); liuxt29@mail2.sysu.edu.cn (X.L.); 2 Institute of Crop Breeding and Cultivation, Shanghai Academy of Agricultural Sciences, Shanghai 201403, China; anpeng0228@163.com

**Keywords:** grain size, grain width, grain length, QTLs, genes, rice

## Abstract

Grain size is a quantitative trait that is controlled by multiple genes. It is not only a yield trait, but also an important appearance quality of rice. In addition, grain size is easy to be selected in evolution, which is also a significant trait for studying rice evolution. In recent years, many quantitative trait loci (QTL)/genes for rice grain size were isolated by map-based cloning or genome-wide association studies, which revealed the genetic and molecular mechanism of grain size regulation in part. Here, we summarized the QTL/genes cloned for grain size and the regulation mechanism with a view to provide the theoretical basis for improving rice yield and breeding superior varieties.

## 1. Introduction

Rice, one of the most important staple foods in the world, is a model plant for studying plant functional genetics. However, it is still urgent to improve rice grain yield with the continuous increase in the world’s population, the deterioration in the environment, and the decrease in the area of arable land. Rice grain yield is composed of three major factors, including effective panicles per plant, grain number per panicle and 1000-grain weight. The 1000-grain weight is affected by grain shape, grain size and the filling of kernels [1]. Grain size, as specified by its three-dimensional structure of seeds (length, width and thickness), is a key determinant of both yield and appearance quality in rice [2]. In recent years, many quantitative trait loci (QTL)/genes for grain size were isolated in rice by map-based cloning and genome-wide association study (GWAS) and revealed the genetic and molecular mechanisms of grain size regulation in part [3,4,5,6,7,8,9,10,11,12,13,14,15,16,17,18,19,20,21,22,23,24]. These QTL/genes were involved in multiple signaling pathways, including G protein signaling, the mitogen-activated protein kinase (MAPK) signaling pathway, the ubiquitin–proteasome pathway, phytohormone signaling, transcriptional regulatory factors, etc. [25,26]. At present, grain size has become an important target trait for the high-yield and high-quality molecular design breeding of rice. Therefore, summarizing the QTL/genes and analyzing the molecular regulatory pathways of grain size can provide an important theoretical foundation for rice breeding.

## 2. Research Progress on the QTL/Genes of Grain Size in Rice

**Figure 1:**
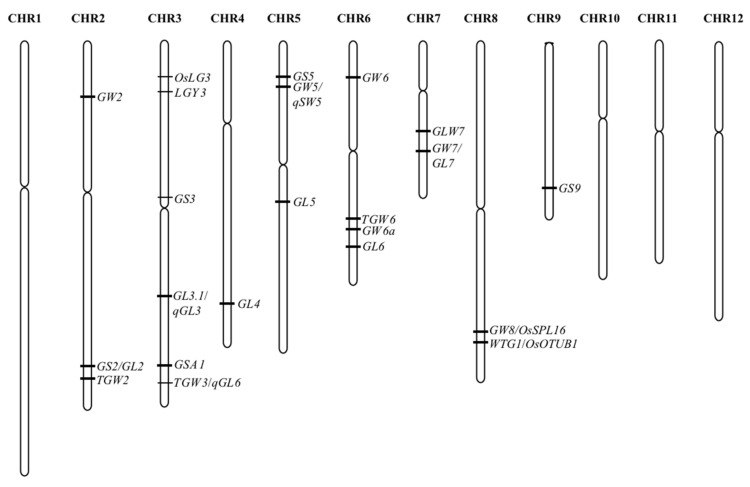
The QTLs isolated for grain size in rice.

Rice grain size is largely determined by a combination of spikelet hulls, the degree of filling and the development of endosperm. It is a quantitative trait, controlled by multiple genes and genetic systems [3]. Therefore, analyzing the QTL and regulatory network is an important method to study grain size in rice. This review addressed only QTL affecting hull size. With the development of rice genome sequencing and functional genomics, more and more functional genes have been gradually resolved. Coupled with cost reduction in the sequencing technologies, a large amount of rice materials have been re-sequenced, and in combination with MutMap and GWAS, the cloning of functional genes related to rice grain size has accelerated, and nearly 200 genes with a direct or indirect role have been cloned. On the other hand, some of the genes were cloned from mutant material with extreme phenotype variation, and it is difficult to meet the actual production demands. Up to now, there are at least 22 grain size-related QTL that have been isolated from natural variation (Figure 1, Table 1). Among these QTLs, most concern grain length and width.

**Table 1:** Identified QTL for grain size in rice.

QTL	Gene ID	Main Regulatory Traits	Signalling Pathways	References
*GW2*	Os02g0244100	Width	Ubiquitin–proteasome pathway	[3]
*GS2*	Os02g0701300	Size	Phytohormone signaling and transcriptional regulatory factor	[12]
*TGW2*	Os02g0763000	Width	Unclear	[23]
*OsLG3*	Os03g0183000	Length	Phytohormone signaling and transcriptional regulatory factor	[27]
*LGY3*	Os03g0215400	Size	Transcriptional regulatory factor	[28]
*GS3*	Os03g0407400	Width	G protein signaling	[4]
*qGL3*	*Os03g0646900*	Size	Phytohormone signaling	[6,8,29]
*GSA1*	Os03g0757500	Size	Phytohormone signaling	[30]
*qTGW3*	Os03g0841800	Size	Phytohormone signaling	[18]
*GL4*	ORGLA04G0254300	Length	Transcriptional regulatory factor	[31]
*GS5*	Os05g0158500	Size	Phytohormone signaling	[5]
*GW5*	Os05g0187500	Width	Phytohormone signaling and ubiquitin–proteasome pathway	[17,26]
*GL5*	Os05g0447200	Length	Phytohormone signaling	[24]
*GW6*	Os06g0266800	Width and length	Phytohormone signaling	[32]
*TGW6*	Os06g0623700	Length	Phytohormone signaling	[9]
*GW6a*	Os06g0650300	Length	transcriptional regulatory factor	[13]
*GL6*	Os06g0666100	Length	unclear	[33]
*GLW7*	Os07g0505200	Length	transcriptional regulatory factor	[16]
*GW7*/*GL7*	Os07g0603300	Width	transcriptional regulatory factor	[14]
*GW8*	Os08g0531600	Width	transcriptional regulatory factor	[14]
*WTG1*	Os08g0537800	Width and thickness	Ubiquitin–proteasome pathway	[34,35]
*GS9*	Os09g0448500	Length	transcriptional regulatory factor	[21]

Note: ORGLA04G0254300 is the gene ID of GL4 in African cultivated rice (*Oryza glaberrima* Steud.).

### 2.1. QTLs Mainly Associated with Grain Length

Grain length refers to the distance from the base of the lowest part of the grain to the longest part. To date, many QTLs related to grain length have been cloned in rice, including *GS3*, *qGL3*, *GL4*, *TGW6*, *GL7*, etc.

*GS3* is the first major QTL identified in rice which regulates grain length and grain weight [36]. This gene encodes a transmembrane protein consisting of five exons. A nonsense mutation in the second exon in all large-grain cultivars resulted in a deletion of 178 amino acids from the C-terminus of the GS3 protein and was accompanied by the early termination of protein translation, indicating that *GS3* negatively regulates grain length and weight [4,36].

*qGL3/GL3.1* encodes a serine/threonine phosphatase belonging to the PPKL family of protein phosphatases which can control rice grain length and yield by directly dephosphorylating substrates to regulate cell cycle proteins [6,8,29]. 

*GL4* controls the grain length on chromosome 4 in African rice (*Oryza glaberrima* Steud.), which regulates longitudinal cell elongation of the outer and inner glumes. Interestingly, *GL4* also controls the seed shattering phenotype like its orthologue *SH4* gene in Asian rice [31].

*TGW6* encodes a novel protein with indole-3-acetic acid (IAA)-glucose hydrolase activity. In sink organs, the Nipponbare *tgw6* allele affects the timing of the transition from the syncytial to the cellular phase by controlling the IAA supply and limiting cell number and grain length. Most notably, loss of function of the Kasalath allele enhances grain weight through pleiotropic effects on source organs, and this leads to significant yield increases [9].

*GL7* is a major QTL that controls rice grain length. It is located on chromosome 7 and encodes a protein homologous to the Arabidopsis LONGFOLIA protein. It can up-regulate the expression level of *GL7* and simultaneously down-regulate the expression level of its neighboring negative factors to make the cells elongated longitudinally, thereby increasing the grain length of rice seeds and changing their appearance quality [14].

*GS5* encodes a serine carboxypeptidase that positively regulates rice grain size [5]. Two key single nucleotide polymorphisms (SNPs) in the *GS5* promoter region cause its differential expression in young spikelets, which determines the differences in grain size. Enhanced expression of *GS5* competitively inhibits the interaction between OsBAK1-7 and OsMSBP1 by occupying the extracellular leucine-rich repeat (LRR) domain of OsBAK1-7, thus preventing OsBAK1-7 from endocytosis caused by interacting with OsMSBP1 [37].

*GLW7* encodes an OsSPL13 protein, which makes the grains larger mainly by increasing cell size. Further population genetic analysis revealed that during the genetic improvement of rice, the large grain gene *GLW7* penetrated from indica to tropical japonica and to a lesser extent temperate japonica through genetic drift, thereby improving the 1000- grain weight and yield of japonica rice [16].

*GS9* encodes a protein without a known conserved functional domain. It regulates grain shape by altering cell division. The *gs9* null mutant has slender grains, while overexpression of *GS9* results in round grains [21].

The rice grain yields QTL *qLGY3*, which encodes the transcription factor OsMADS1 and contains the MADS domain, and is a key effector in the downstream of the G protein βγ dimer. The variable splicing protein OsMADS1 ^lgy3^ produces longer grains and improves the quality and yield of rice grains [28]. *OsLG3* is a member of ERF family transcription factor, and it can positively regulate rice grain length with no effect on grain quality [27].

Loss of function at the QTL *qGL5* (*OsAUX3*) could lead to more significant grain length and weight. Research showed that transcription factor OsARF6 binds directly to the auxin response elements of the *OsAUX3* promoter and controls grain length by altering longitudinal expansion and auxin distribution/content in glume cells. Moreover, *miR167a* positively regulates grain length and weight by directing *OsARF6* mRNA silencing. These results indicated that a novel *miR167a*-*OsARF6*-*OsAUX3* module regulates grain length and weight, providing a potential target for the improvement of rice yield [24].

### 2.2. QTLs Mainly Associated with Grain Width 

Grain width refers to the distance between the widest parts on both sides of the inner and outer glume, which is one of the main grain shape traits of rice. Cloning the QTLs related to its growth and development is particularly important for studying rice yield and appearance quality. Currently, there are not many cloned QTLs for grain width in rice, mainly *GW2*, *GW5*, *GW7*, *GW8*, *TGW2*, etc.

*GW2* is the first grain width QTL cloned in rice. It is located on the short arm of chromosome 2 and encodes a ubiquitin ligase that negatively regulates cell division by anchoring substrates to the proteasome for degradation [3]. The mutation of this gene could not recognize the substrate which should be degraded, thus it activated the division of the glume shell cells and increased the width of the glume shell. This indirectly increased the filling rate and consequently expanded the size of the endosperm, and ultimately increased glume shell width, grain weight and yield [3].

*GW5* encodes a calmodulin binding protein [11], as studies have shown that *GW5* can interact with polyubiquitin, suggesting that it may regulate grain width and weight via the ubiquitin proteasome pathway [38].

*OsSPL16*/*GW8* is a transcription factor containing an SBP structural domain, which is able to bind directly to the *GW7* promoter and repress its expression, and then regulate rice grain width [14].

*GW6* encodes a GA-regulated GAST family protein and positively regulates grain width and weight. It is highly expressed in the young panicle and increases grain width by promoting cell expansion in the spikelet hull. Knockout of *GW6* exhibits reduced grain size and weight, whereas overexpression of *GW6* results in increased grain size and weight [32]. 

*TGW2* encodes the cell number regulator 1(OsCNR1). The TGW2 protein interacts with the KRP1 protein, which regulates cell cycle and affects cell proliferation and expansion in glumes, and negatively regulates grain width and grain weight in rice [23].

### 2.3. QTLs Mainly Associated with Thickness 

Grain thickness refers to the distance between the thickest parts on both sides of the inner and outer glume. Few studies have been reported on rice grain thickness. Most researchers agree that thickness is a quantitative trait which is also regulated by multiple genes.

*WTG1* encodes a deubiquitinating enzyme with homology to human OTUB1 and is a functional deubiquitinating enzyme [34]. The *wtg1-1* mutant exhibits wide, thick, short and heavy grains and also shows an increased number of grains per panicle. Corresponding, overexpression of *WTG1* results in narrow, thin, long grains [35].

## 3. Molecular Regulatory Networks of Grain Size in Rice

**Figure 2:**
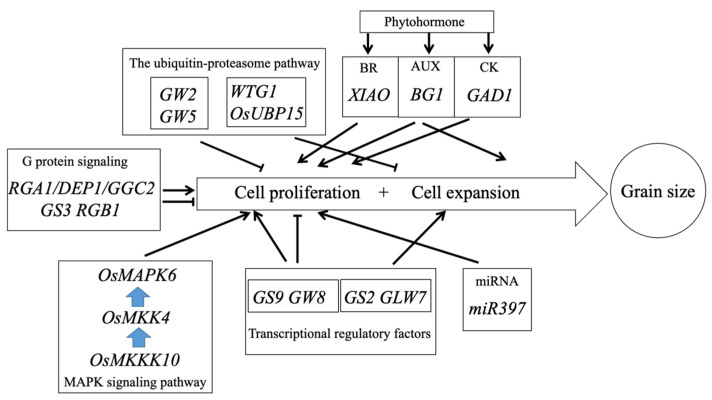
The grain size control in rice.

Rice grain size is restricted by the size of the spikelet hull [39], which is determined by both cell proliferation and expansion (Figure 2). The regulation of grain size involves a complex genetic network that begins with cell proliferation and ends with the completion of grain filling [39,40]. In rice, the signaling pathways regulating grain size are relatively conservative, and a summary analysis of the reported grain size-related genes shows that these regulatory pathways include G protein signaling, the mitogen-activated protein kinase (MAPK) signaling pathway, the ubiquitin–proteasome pathway, phytohormone signaling, transcriptional regulatory factors, etc. [25,26]. It has been shown that there are also interactions between the different grain size regulatory pathways [41].

### 3.1. Control of Grain Size by G Protein Signaling

G protein exists in plants as a heterotrimer consisting of α, β, and γ subunits which can regulate multiple signaling pathways [42]. The loss-of-function mutants of rice G protein α subunit D1 are less sensitive to BR, indicating that D1-mediated heterotrimeric G protein and BR signal transduction pathways co-regulate grain size in rice [43,44,45,46]. In addition, rice yield QTL-*qLGY*, which encodes a transcription factor OsMADS1, containing a MADS domain, is a key effector downstream of the G protein βγ dimer. The alternative splicing protein OsMADS1 ^lgy3^ produces slender grains, which not only increases rice yield, but also improves rice quality. GS3 and DEP1 are the Gγ subunits in rice. They can directly interact with MADS transcription factors, and at the same time can compete with each other for Gβ subunits to regulate rice grain size [28]. 

### 3.2. Control of Grain Size by the Mitogen-Activated Protein Kinase (MAPK) Signaling Pathway

Mitogen-activated protein kinase (MAPK) is a component of a series of intracellular cascades that can respond to a variety of extracellular stimuli [47]. MAPK signaling is involved in many aspects of plant growth and development, and MAPK phosphatase (MKP) specifically removes the phosphate group from activated MAPK, thereby inactivating it. *LARGE8* negatively regulated rice grain size by directly interacting with and inactivating OsMAPK6, resulting in fewer glume cells [48]. Then, the study of the loss-of-function mutants and overexpression lines of *OsMKKK10* and the gain-of-function mutants of *OsMKK4* indicated that the *OsMKKK10*-*OsMKK4*-*OsMAPK6* signaling pathway positively regulates grain size and weight in rice [49].

In addition, rice grain size is mostly regulated by multiple signaling pathways. DSG1 can connect the MAPK pathway and the BR pathway to regulate its endogenous BR content and grain size [50].

### 3.3. Control of Grain Size by the Ubiquitin–Proteasome Pathway

Currently, the ubiquitin pathway has received widespread attention for its involvement in the formation of plant seed size [51,52,53]. Ubiquitin is a conserved protein with 76 amino acids which can bind to the target protein through the covalent bond of ubiquitin-activating enzymes(E1s), ubiquitin-conjugating enzymes(E2s) and ubiquitin ligases(E3s) [54,55]. The process of ubiquitination is that a ubiquitin-activating enzyme recognizes and activates ubiquitin molecules with ATP providing energy, and the ubiquitin-conjugating enzyme (E2) connects the activated ubiquitin, and then the ubiquitin protein ligase (E3) recognizes the target protein and promotes the transfer of the ubiquitin which linked to E2 to the target protein. When the ubiquitin concentration reaches a certain level, the target protein will be degraded by the 26S proteasome [56]. The *GW2* gene, which encodes an E3 ubiquitin ligase, negatively regulates glume cell division and affects rice grain width, grain weight and yield [3]. Moreover, the E3 ubiquitin ligase GW2 ubiquitinates WG1 and targets it for degradation; WG1 interacts with the transcription factor OsbZIP47 and represses its transcriptional activity by associating with the transcriptional co-repressor ASP1, indicating that WG1 may act as an adaptor protein to recruit the transcriptional co-repressor [57]. The *HGW* gene, which positively regulates cell proliferation, is co-expressed with genes in the ubiquitin pathway. Studies have found that the protein encoded by *HGW* contains ubiquitin-related domains, indicating that it can regulate the heading date and grain weight through the ubiquitin pathway in rice [58]. *LARGE2* encodes a HECT-domain E3 ubiquitin ligase OsUPL2 and regulates panicle size and grain number in rice [59].

### 3.4. Control of Grain Size by Phytohormone Signaling

Plant hormones are important regulators of grain size in rice [60]. Brassinosteroids (BR), cytokinin (CK) and auxin (AUX) are three main hormones that regulate rice grain shape.

BR can affect the grain filling rate and grain size of rice by regulating the expression of related genes, thereby affecting rice yield [61,62,63]. Several BR synthesis-related genes have been reported in rice, and mutations in BR genes with change in grain size is often accompanied by plant height and leaf angle alteration, such as *D2* [64,65], *D11* [66,67], *OsBSK3* [68], *SLG* [64], *XIAO* [69], *GS2* [11,12,61], *OML4* [70], etc. 

In higher plants including rice, auxin can affect the filling rate and grain size [50]. The auxin-responsive gene *BG1* can positively regulate the response and transport of auxin, thereby promoting the elongation and expansion of glume cells, and regulating the grain size in plants [50]. The gene *OsARF19* is induced by both auxin and BR, and it can bind to the promoter of the BR receptor gene *OsBR11*, which directly affects the expression of *OsBR11*, while overexpression of *OsARF19* can cause plants to exhibit short, narrow leaves, small grains, etc [8]. *qGL5* encodes the transmembrane amino acid transporter OsAUX3 which can regulate grain length and weight in rice by *miR167a*-*OsARF6*-*OsAUX3* module [24]. *ERECTA1* (*OsER1*), a negative regulator of spikelet number per panicle, which acts upstream of the *OsMKKK10*-*OsMKK4*-*OsMPK6* cascade, and the *OsER1*-*OsMKKK10*-*OsMKK4*-*OsMPK6* pathway is required to maintain cytokinin homeostasis [71]. 

Cytokinins are mainly distributed in young plant tissues, such as root meristems, young leaves, and fruits. They positively regulate cell division and proliferation in plant apical meristems [72]. The gene *GAD1* encodes the epidermal patterning factor-like family (EPFL1), which can regulate rice grain length, the number of spikelets per panicle and the development of awn by reducing the content of plant endogenous CK [73].

### 3.5. Control of Grain Size by Transcriptional Regulatory Factors

Transcriptional regulatory factors that regulate gene expression include zinc finger, helix-loop-helix, SPL, MYB, etc., which can widely affect the development of plant cells [74,75]. *GLW7* encodes the plant transcription factor SPL13, which positively regulates the size of glume cells and increases rice yield by increasing grain length and 1000-grain weight [16]. *GW8* is a transcription factor containing the SBP domain, which regulates rice grain width and can directly bind to the *GW7* promoter and inhibit its expression [14]. *GS2*, which encodes Growth-Regulating Factor 4 (OsGRF4), a transcriptional regulator, regulates grain size by promoting cell division and cell expansion [12]. The rice transcription factor *OsWRKY53* has been reported as a new target gene of OsGSK2. OsGSK2 phosphorylates OsWRKY53, reduces its stability and negatively regulates the growth of rice seeds [76].

### 3.6. Control of Grain Size by Other Pathways

In recent years, studies have shown that miRNA also has a regulatory effect on rice grain size. The *miR1848* in rice can reduce the transcription level of the *OsCYP51G3* to affect the biosynthesis of BR and make rice seeds smaller [77]. *miR397* can suppress the expression of *OsLAC* and promote the transduction of the BR signal, thereby increasing rice yield [78].

Epigenetic modifications can affect the expression of genes, which can lead to abnormal phenotypes in plants. The genomic imprinting of endosperm can regulate its development by inhibiting the expression of related genes, thereby regulating the rice grain size [79,80]. Hypomethylation in the promoter region of *RAV6* gene leads to small grain in *Epi-rav6* mutants, indicating that methylation also plays an important role in the regulation of grain size [81]. The reduction in rice spikelets per panicle of the *SPL14* allele indicates that DNA methylation and histone modification can regulate the expression of *SPL14* [43].

Some genes that regulate seed germination can also affect rice grain size. Phenotypic analysis of *OsSPMS1* RNA interference and overexpression lines showed that this gene negatively regulates rice seed germination, grain size and yield [82].

## 4. Discussion and Perspective

With the continuous increase in the population, the deterioration in environment, and the decrease in arable land area, it is still urgent to improve the grain yield of rice, one of the most important staple foods. Rice grain size is closely related to the yield. However, due to the complicated genetic background of rice itself, there are still many obstacles in the study of grain size. Although many genes related to grain shape have been reported, in fact only a small part can be applied to production application. Thus, it is necessary to speed up the cloning of grain shape-related genes. Currently, re-sequenced and in combination with MutMap and GWAS can be used to mine more grain size genes and provide abundant genetic resources for rice breeding. In addition, the interaction between most cloned grain size genes and the regulatory network is not clear. For example, the bridge between the MAPK and BR signaling pathways is also not clear. Therefore, it is necessary to further explore the molecular mechanisms regulating rice grain size, thereby providing rich genetic resources and a theoretical basis for high yield and quality breeding.

The ultimate goal of studying grain size genes is for breeding. From a breeding point of view, marker-assisted selection (MAS) is a more acceptable method than the transgenic approaches. It is also clear that multiple genes are needed to improve rice grain size [83,84]. To date, many gene-linked markers have been developed to facilitate MAS breeding [85]. The PCR-based molecular markers set covered the main cloned QTLs for grain size and proved the effects of these markers in discriminating grain size traits, and it may facilitate both association and linkage analyses for future genetic study, and provides efficient tools for the rational design of grain size as part rice breeding strategies in the future [86]. In addition, efficient genome editing using CRISPR technology, aggregating favorable alleles, editing negatively regulating grain size genes and targeted knockout and accurately improving individual bad traits in varieties will be sped up breeding.

## Data Availability

All of the data generated or analyzed during this study are included in this published article.
